# Thrombin-activated platelet-rich plasma enhances osteogenic differentiation of human periodontal ligament stem cells by activating SIRT1-mediated autophagy

**DOI:** 10.1186/s40001-021-00575-x

**Published:** 2021-09-15

**Authors:** Yunhe Xu, Xiaoning Wang, Wenshu Liu, Weiwei Lu

**Affiliations:** 1 Department of Stomatology, The First Hospital of Jilin University, Changchun, Jilin, 130021 China; 2 Department of Blood Transfusion, The First Hospital of Jilin University, No. 71 Xinmin Street, Chaoyang District, Changchun, 130021 Jilin China

**Keywords:** Autophagy, Osteogenic differentiation, Periodontal ligament stem cells, Platelet-rich plasma, Viability

## Abstract

**Background:**

Platelet-rich plasma (PRP) has the potential to be used for bone regeneration. However, its effect on osteogenic differentiation of human periodontal ligament stem cells (hPDLSCs) and its effect on cell autophagy of hPDLSCs remain unknown. In this study, we investigated the effects of PRP on cell viability and osteogenic differentiation of hPDLSCs and the underlying molecular mechanisms.

**Methods:**

hPDLSCs were isolated and identified by morphology and flow cytometry analysis. Next, thrombin-activated PRP was used to stimulate hPDLSCs. The MTT assay was used to analyze cell viability. Osteogenic differentiation was investigated using alkaline phosphatase (ALP) activity assay, alizarin red S (ARS) staining, and gene expression analysis of osteogenic markers. Expression of the autophagic proteins was determined using western blotting.

**Results:**

Thrombin-activated PRP significantly enhanced cell viability, ALP activity, osteogenic-related mRNA levels and alizarin red-mineralization activity in hPDLSCs in a dose-dependent manner. Furthermore, activated PRP dose-dependently increased LC3-II/I ratio and the expression of SIRT1 and Beclin-1. PRP treatment also enhanced the autophagic flux. It was also demonstrated that the inhibition of SIRT1 using sirtinol or suppression of autophagy by 3-methyladenine (3-MA) abrogated PRP-induced viability and osteogenic differentiation of hPDLSCs.

**Conclusion:**

Our study suggested that thrombin-activated PRP accelerated the viability and osteogenic differentiation of hPDLSCs via SIRT1-mediated autophagy induction.

## Highlights

1. PRP enhances cell viability and osteogenic differentiation of hPDLSCs.
2. Activated PRP dose-dependently increases cell autophagy.
3. Inhibition of autophagy abrogates PRP-induced osteogenic differentiation of hPDLSCs.

## Introduction

Periodontal ligament stem cells (PDLSCs) are multipotent mesenchymal stem cells (MSCs) derived from periodontal tissues, with the potentials of high proliferation, self-renewal and multidirectional differentiation [1]. Since PDLSCs are convenient to obtain and easy to culture, PDLSCs are considered to be suitable cell sources for periodontal regeneration [2]. Numerous studies demonstrated that PDLSCs have the capacity to differentiate into osteoblasts, chondrocytes, myocytes, adipocytes, and neuron-like cells in vitro [3, 4]. Besides, PDLSCs have been proposed as alternative stem cells for periodontal regenerative therapy due to the osteogenic differentiation potential.

Platelet-rich plasma (PRP), a platelet concentrate extracted from autologous blood by centrifugation, was originally used in the clinic to improve hemostasis [5]. Upon activation, PRP releases a large number of growth factors, such as platelet-derived growth factor (PDGF), transforming growth factor beta (TGF-β), insulin-like growth factor (IGF), epidermal growth factor (EGF) and vascular endothelial growth factor (VEGF), which plays a crucial role in bone regeneration due to its potential to repair tendons, ligaments, skeletal muscles and cartilage [6, 7]. Among the methods, thrombin is a common and widely used approach to activate PRP. It was found that thrombin-activated PRP could improve the fat graft survival in nude mice and could be used as a substitute for fetal bovine serum in the engineering of osteogenic/vasculogenic grafts [8, 9]. In recent years, several researches have proposed that activated PRP can induce proliferation and osteogenic ability of bone marrow- and adipose-derived mesenchymal stem cells and skeletal muscle satellite cells [10, 11]. However, the involvement of PRP in the proliferation and osteogenic differentiation of PDLSCs has not been fully clarified.

Recently, the role of autophagy is noticed in osteogenesis. Generally, it is considered that activation of autophagy may promote osteogenesis. It was reported that the activation of autophagy by titanium could promote osteogenesis through regulating YAP and β-catenin signaling [12]. The activation of autophagy both promote osteogenesis and is beneficial for cell proliferation of mesenchymal stem cells and may lead to cell senescence [13]. On the contrary, the inhibition of autophagy might lead to suppression of osteogenesis [14]. Besides, it was found that resveratrol could promote osteogenesis by enhancing SIRT1-mediated autophagy in osteoporosis rats [15]. Additionally, PRP is also found to increase autophagy in human osteoarthritic cartilage [16]. However, whether PRP can influence the osteogenesis of PDLSCs through regulation of autophagy is unclear.

In the present study, we hypothesized that PRP might have positive effects on PDLSC’s viability and differentiation. We investigated the effects of activated PRP on the cell viability and the osteogenic differentiation of hPDLSCs and the underlying molecular mechanisms. The results of the present study might provide deeper understanding for the potential application of hPDLSCs in periodontal regeneration.

## Materials and methods

### Third molars and peripheral venous blood samples

The present study was conducted with the approval of the Ethics Committee of The First Hospital of Jilin University (Approval No. JLFH2020-048). Third molars and peripheral venous blood were obtained from 10 patients (5 males, 5 females; 25–28 years old) who underwent surgical removal of impacted mandibular third molar teeth for orthodontic treatment after obtaining informed consent. The inclusion criteria of these patients were: (1) all patients with no history of systemic diseases and periodontal diseases; (2) all patients who received routine blood test and no abnormal indices like elevated inflammatory factors or abnormal platelet counts were found; (3) all participants were non-smokers. Other patients who did not meet the above criteria were excluded, including patients with periodontal diseases, inflammation, dysfunction of coagulation, or other severe renal, cardiovascular, liver diseases. All participants were with normal platelet counts within 231 ± 63 × 10^9^/L.

### Preparation of activated PRP

PRP was obtained using a two-step centrifugation method described by Landesberg et al. [17]. Briefly, fasting peripheral venous blood samples (50 mL) were collected in tubes containing EDTA anticoagulant, and were centrifuged for 10 min at 2000×*g*. The yellow plasma containing platelets, leukocytes, and some erythrocytes was then centrifuged for another 10 min at 2000×*g*. Then the supernatant was removed and the platelets accumulated in the thrombocyte pellet were used as PRP. Then, a 9:1 (v/v) mixture of PRP and solution containing 10% CaCl_2_ solution and 1000U bovine thrombin (Sigma-Aldrich, St. Louis, MO, USA) was incubated for 30 min at room temperature. Activated PRP was centrifuged at 4000×*g* for 5 min and the supernatant was filtered through a 0.22-μm membrane. The platelet count was then tested within 950 ± 219 × 10^9^/L, > 4-fold of the whole blood platelet. The samples were then stored at − 20 °C.

### Cell isolation and culture

The teeth were rinsed with serum-free PBS containing 100 U/mL penicillin and 100 mg/mL streptomycin (Gibco, Carlsbad, CA, USA). The periodontal ligament (PDL) was removed from the middle one-third of the tooth root, washed with PBS and centrifuged for 5 min at 1000×*g*. After digestion in 3 g/L collagenase type I and 4 g/L dispase for 1 h, the single-cell suspension was cultured in α-MEM (Thermo Fisher Scientific, Waltham, MA, USA) supplemented with 10% fetal bovine serum (FBS), 100 U/mL penicillin and 100 μg/mL streptomycin (Gibco) in a humidified atmosphere of 5% CO_2_ at 37 °C. The morphology of hPDLSCs was observed using an optical microscope at different time points of culturing after 7 days, 10 days and 14 days. Cells were characterized by surface markers, positive for CD146 and STRO and negative for CD34 by flow cytometry analysis upon reaching 70% confluence.

Then, hPDLSCs were incubated in the above α-MEM (Thermo Fisher Scientific, Waltham, MA, USA) containing 10% FBS, 100 U/mL penicillin and 100 μg/mL streptomycin (Gibco), which were supplemented with different concentrations of activated PRP (5%, 10% and 20%). Briefly, hPDLSCs were seeded in 96-well culture plates containing 100 μL culture medium with activated PRP (5%, 10% and 20%). The culture medium was replaced every 2 days. For inhibition of SIRT1 or autophagy, cells were cultured with 10 µM SIRT1 inhibitor sirtinol [18, 19] or 10 mM autophagy inhibitor 3-methyladenine (3-MA) [20, 21], purchased from Sigma-Aldrich (St. Louis, MO, USA) for another 24 h. To inhibit the function of lysosome, cells were treated with lysosomal inhibitor Bafilomycin A1 (BFA, 200 nM, Sigma-Aldrich) for 24 h.

### 3-(4,5-Dimethylthiazole-2-yl)-2,5-biphenyl tetrazolium bromide (MTT) assay

Before co-culture and after co-culture for 24 h, 48 h, 72 h and 96 h with different concentrations of PRP, hPDLSCs were re-seeded into 96-well microplates and incubated with MTT for 4 h. Supernatants were discarded and the absorbance was measured at 570 nm after the addition of dimethyl sulfoxide (DMSO) on an automatic microplate reader (Dynex Technologies, Gentilly, VA, USA). Cell viability was defined relative to untreated control cells. Each experiment was performed in triplicate and repeated at least three times.

### Alkaline phosphatase (ALP) activity assay

After 0-, 1-, 3-, 5- and 7-day treatment of different concentrations of PRP, the ALP activity was measured. Briefly, cells were washed twice with cold PBS and lysed in Triton X-100. The ALP activity was then evaluated using an ALP activity kit (Jian Cheng Biotech, Nanjing, China) strictly according to the manufacturer’s instruction. Protein concentration was quantified with a BCA protein assay kit (Beyotime, Shanghai, China). The absorbance was measured with a microplate reader at the wavelength of 520 nm.

### RNA extraction and qRT-PCR analysis

After 7-day treatment, the mRNA expression of collagen type I (COL-I), osteopontin (OPN), runt-related protein 2 (Runx2) and osteocalcin (OCN) was measured. Total RNA was extracted using Trizol reagent (Invitrogen, Carlsbad, CA, USA), and reverse-transcribed to cDNA using a MiRcute miRNA First-strand cDNA synthesis kit (Tiangen Biotech, Beijing, China) according to the manufacturer’s protocol. Relative mRNA expression levels of the osteogenic markers COL-I, OPN, Runx2 and OCN were detected using the MiRcute miRNA qPCR detection kit (Tiangen Biotech) performing on an ABI 7500 real-time PCR system (Applied Biosystems, Carlsbad, CA, USA) and calculated by the 2^−ΔΔCt^ method. GAPDH was used as an internal control. The primer sequences used were as follows: COL-I, forward 5′-AGAACAGCGTGGCCT-3′, reverse 5′-TCCGGTGTGACTCGT-3′; OPN, forward 5′-CTGAACGCGCCTTCTGATTG-3′, reverse 5′-ACATCGGAATGCTCATTGCTCT-3′; Runx2, forward 5′-GGAGTGGACGAGGCAAGAGTTT-3′, reverse 5′-AGCTTCTGTCTGTGCCTTCTGG-3′; OCN, forward 5′-GGCAGCGAGGTAGTGAAGAG-3′, reverse 5′-GATGTGGTCAGCCAACTCGT-3′; GAPDH, forward 5′-CGCTCTCTGCTCCTCCTGTT-3′, reverse 5′-CCATGGTGTCTGAGCGATGT-3′.

### Alizarin red S (ARS) staining

After 21-day treatment of PRP, cells were fixed in 4% paraformaldehyde for 30 min and stained with 2% ARS solution (Sigma-Aldrich) for 5 min. The stained cells were desorbed with 10% cetylpyridinium chloride (Sigma-Aldrich), observed under an inverted optical microscope and photographed using a digital camera.

### Western blot

The protein levels of SIRT1, LC3 II/I and Beclin-1 were also measured after 7-day treatment. Total protein was extracted by lysis buffer containing phenylmethanesulfonyl fluoride (PMSF) and centrifuged at 10,000×*g* for 20 min. Aliquots of 20 μg protein from each sample were blended with loading buffer, denatured in boiling water for 5 min, separated with 10% sodium dodecyl sulfate polyacrylamide gel electrophoresis (SDS-PAGE) and were electro-transferred to polyvinylidene fluoride (PVDF) membranes. The samples were then blocked with 5% skim milk at room temperature for 2 h, followed by eluted with Tris-buffered saline Tween (TBST) for 6 times. The membranes were probed with primary antibodies against SIRT1, LC3-I, LC3-II, Beclin-1 and GAPDH (1:1000; Cell Signaling Technology, Boston, MA, USA) overnight at 4 ℃. Then blots were incubated with horseradish peroxidase (HRP)-conjugated secondary antibodies (1:1000 dilution; Cell Signaling Technology, Boston, MA, USA) at room temperature for 2 h after washed with TBST. The protein levels were quantified using enhanced chemiluminescence (Thermo Scientific, Shanghai, China).

### Statistical analysis

Statistical analysis of all data was performed using SPSS version 22.0 (IBM, Chicago, IL, USA). All results were presented as the mean ± standard deviation (SD). Comparison among three or more groups was performed by one-way analysis of variance (ANOVA) followed by Tukey post hoc test and comparison between two groups was made by two-sided Student’s *t* test. *P* < 0.05 was taken as statistically significant.

## Results

### The characterization of hPDLSCs

**Fig. 1:**
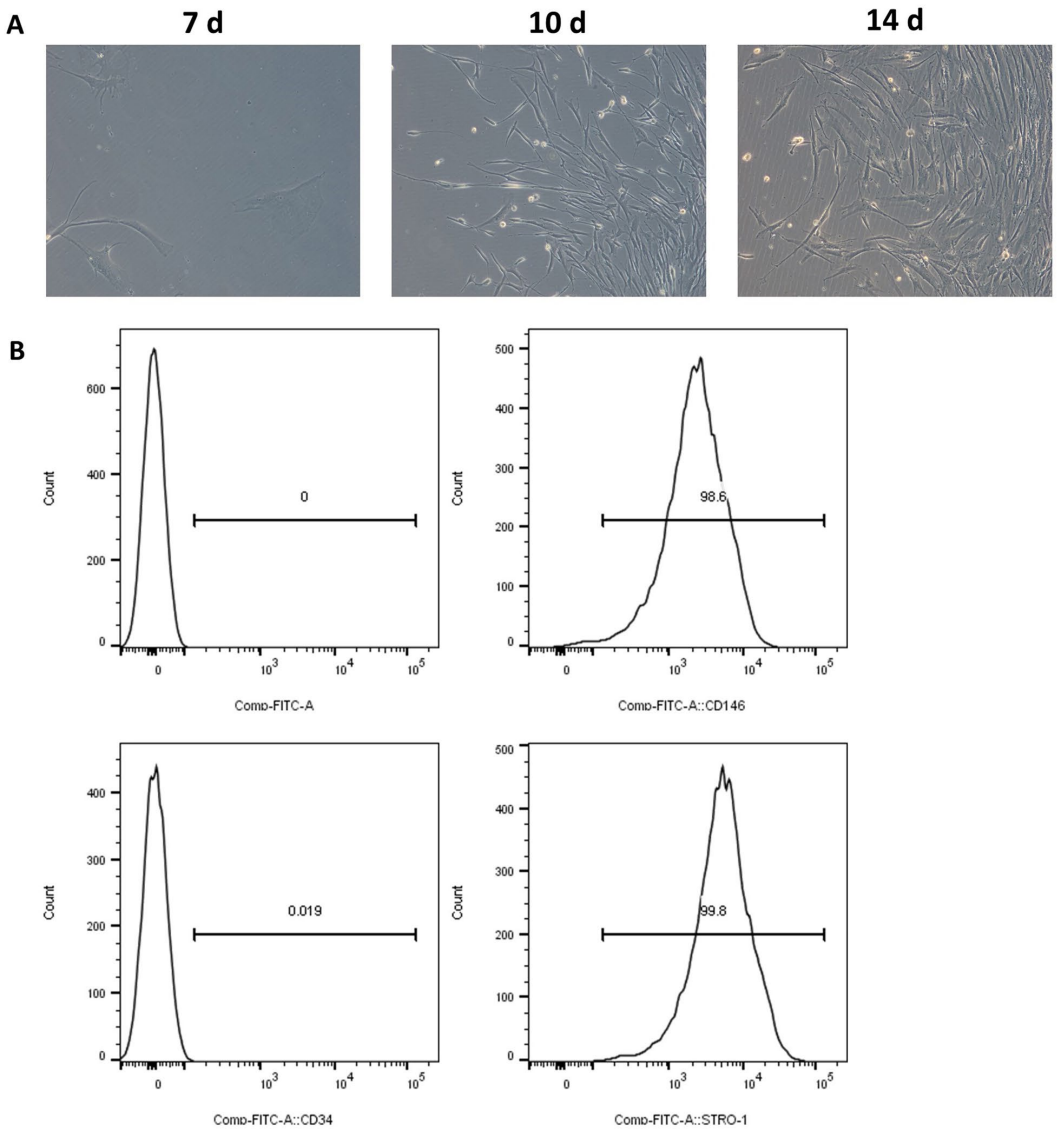
The characterization of hPDLSCs. **A** After isolation, hPDLSCs exhibited a spindle-shaped morphology. **B** By FCM analysis, hPDLSCs were positive for MSCs markers CD146 and STRO-1, while negative for hematopoietic cells markers CD34 and CD45

The primary cultures of hPDLSCs were analyzed at the third passage. The cellular morphology was observed by an optical microscope, showing that the cells appeared spindle-shaped with ovoid nuclei (Fig. 1A). As shown in Fig. 1B, the flow cytometry analysis showed the phenotypes of purified cells were uniformly positive for MSC-related markers CD146 and STRO-1, and negative for hematopoietic stem cell marker CD34, which were consistent with the characteristics of hPDLSCs [22].

### Activated PRP enhances the cell viability and osteogenic differentiation of hPDLSCs

**Fig. 2:**
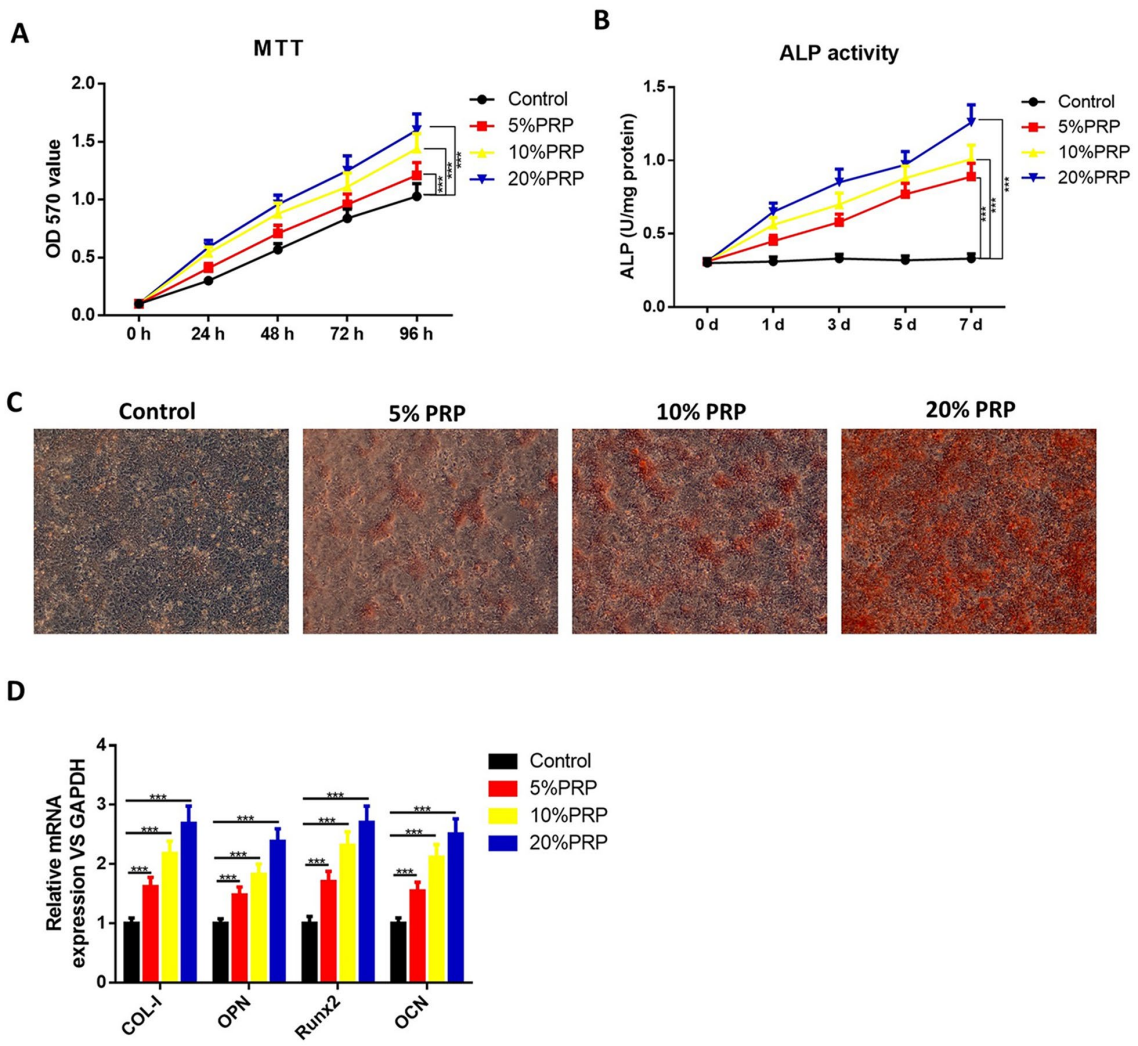
Effects of activated PRP on the proliferation and osteogenic differentiation of hPDLSCs. hPDLSCs were treated with or without thrombin-activated PRP. **A** MTT assay results of hPDLSC proliferation. **B** ALP staining of PRP-treated hPDLSCs. **C** QRT-PCR results showed the expression of COL-I, OPN, Runx2 and OCN. GAPDH was used as an internal control. **D** ARS staining was performed to examine calcium deposits. ^***^P < 0.001

To determine the potential effects of PRP on cell viability and osteogenic differentiation, we treated hPDLSCs with 5%, 10% and 20% thrombin-activated PRP. MTT results showed that exposure to activated PRP significantly stimulated the viability of hPDLSCs compared to the control group in a dose-dependent manner (Fig. 2A). ALP activity was also measured in the presence of activated PRP on days 1, 3, 5 and 7. As shown in Fig. 2B, ALP activity was significantly increased by the treatment of PRP. On day 21 after treatment, we performed ARS staining to examine calcium deposits. The results showed that activated PRP induced extracellular matrix calcification of hPDLSCs (Fig. 2C). Meanwhile, we performed qRT-PCR to detect the mRNA levels of osteogenic-related factors, including COL-I, OPN, Runx2 and OCN and observed a higher expression of COL-I, OPN, Runx2 and OCN in PRP-treated groups after 7-day treatment (Fig. 2D).

### Activated PRP promotes the autophagy of hPDLSCs

**Fig. 3:**
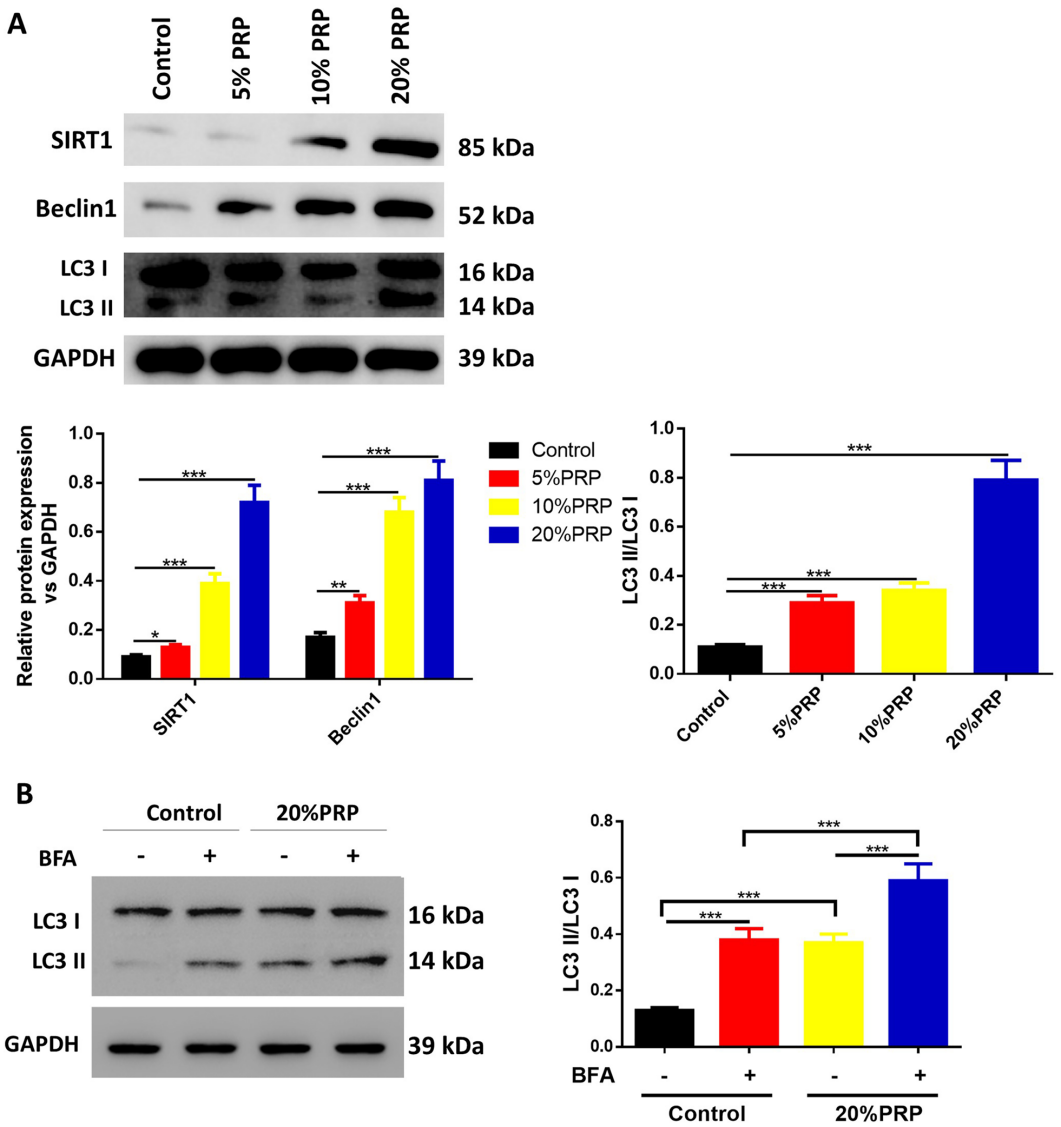
Effects of activated PRP on the autophagy of hPDLSCs. hPDLSCs were treated with or without thrombin-activated PRP. **A** Western blotting analyses demonstrated the expression of SIRT1, LC3-I, LC3-II and Beclin-1. GAPDH was used as an internal control and the quantitative analysis. **B** Autophagic flux was determined by treatment of Bafilomycin A1 in control cells or cells treated with 20% PRP and protein levels of LC3 II/I and Beclin-1 were measured. ^*^P < 0.05, ^**^P < 0.01

We next analyzed the effects of PRP on the autophagy of hPDLSCs by assessing the expression of autophagy-related proteins, such as SIRT1, LC3-I, LC3-II and Beclin-1. Figure 3A shows that activated PRP observably increased the protein levels of SIRT1 and Beclin-1, as well as the ratio of LC3-II/LC3-I. Additionally, autophagic flux was detected by treatment of Bafilomycin A1 in control cells or cells treated with 20% PRP. It was found in both groups that the treatment of Bafilomycin A1 markedly enhanced the ratio of LC3-II/LC3-I and when treated with 20% PRP, the ratio of LC3-II/LC3-I also significantly increased when compared with the control group (Fig. 3B).

### Autophagy is involved in activated PRP-induced hPDLSC viability and osteogenic differentiation

**Fig. 4:**
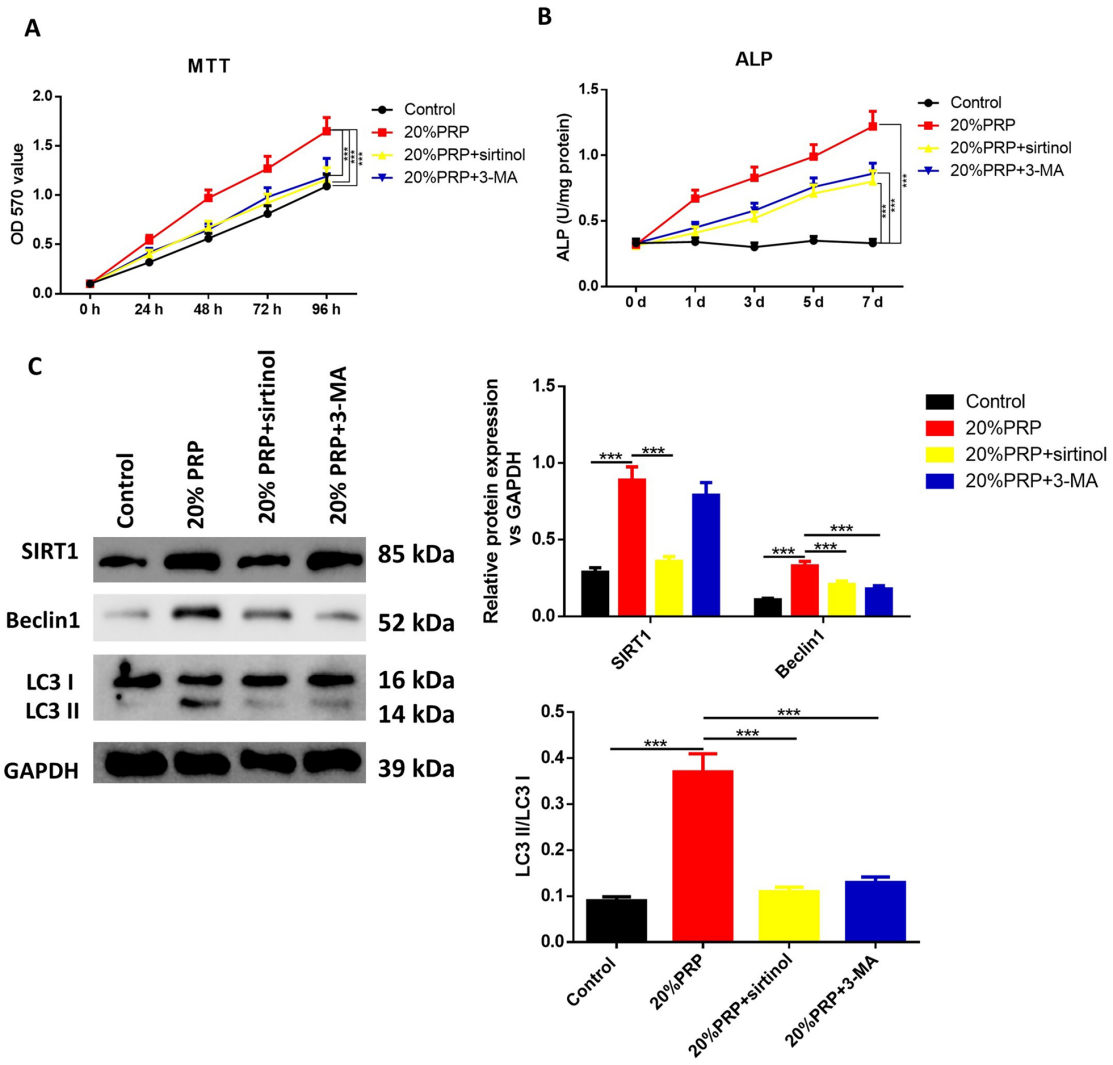
Effects of autophagy inhibition on activated PRP-induced hPDLSC proliferation and ALP activity. hPDLSCs were treated with 20% thrombin-activated PRP or together with sirtinol or 3-MA. **A** MTT assay results of hPDLSC proliferation. **B** ALP staining of PRP-treated hPDLSCs. **C** Western blotting analyses and quantitative analysis demonstrated the expression of SIRT1, LC3-I, LC3-II and Beclin-1. GAPDH was used as an internal control. *P < 0.05, **P < 0.01, ***P < 0.001

**Fig. 5:**
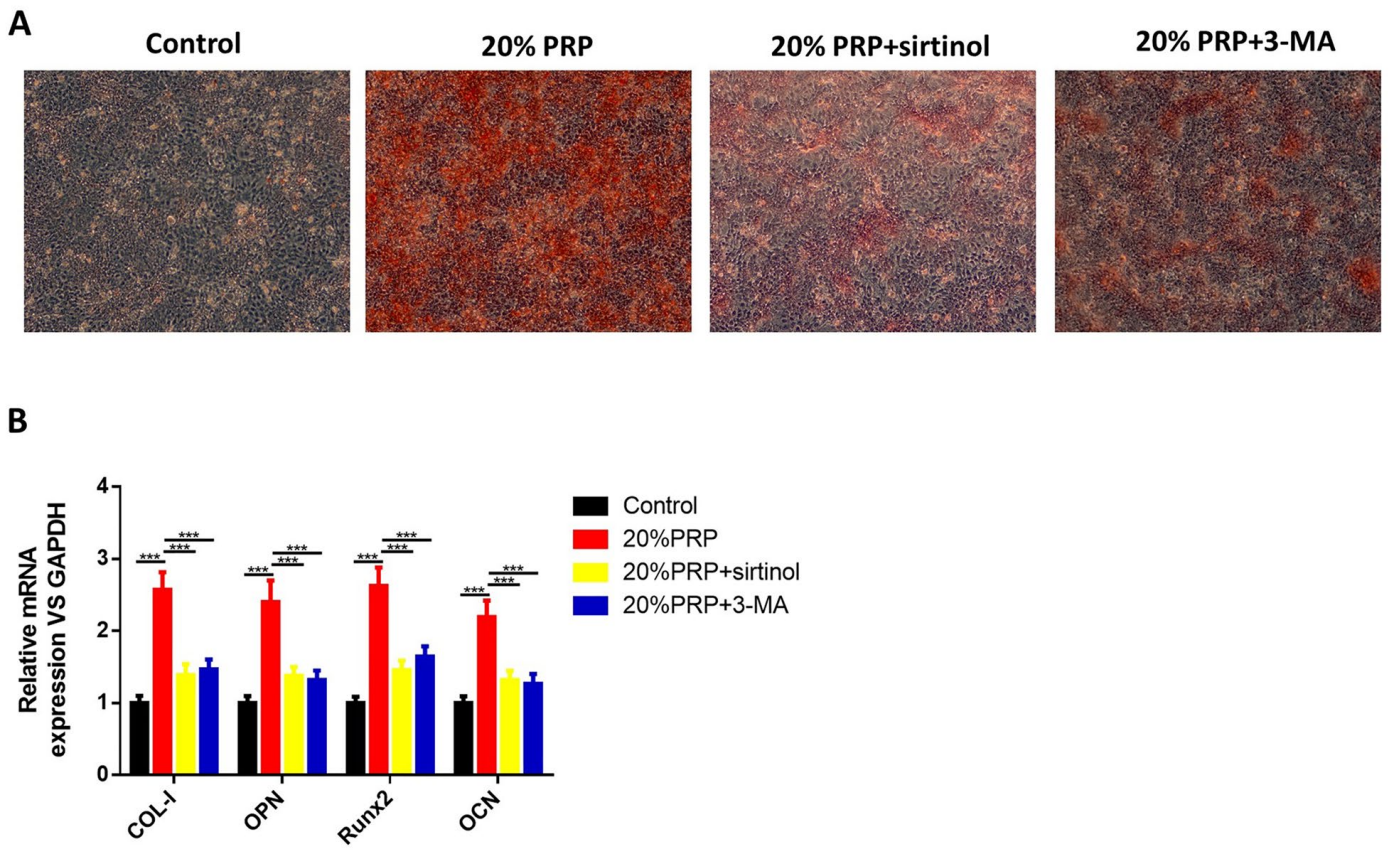
Effects of autophagy inhibition on activated PRP-induced hPDLSC osteogenic differentiation. hPDLSCs were treated with 20% thrombin-activated PRP or together with sirtinol or 3-MA. **A** ARS staining was performed to examine calcium deposits. **B** QRT-PCR results showed the expression of COL-I, OPN, Runx2 and OCN. GAPDH was used as an internal control. ***P < 0.001

The involvement of autophagy in the viability and osteogenic differentiation induced by PRP was further validated by the SIRT1 inhibitor sirtinol or the autophagy inhibitor 3-MA. It was shown that sirtinol or 3-MA markedly inhibited PRP stimulation-induced increase of cell viability (Fig. 4A). Activated PRP-induced elevation of ALP activity was also significantly decreased by treatment of sirtinol or 3-MA (Fig. 4B). Western blots (Fig. 4C) showed that the effects of PRP on autophagy-related proteins (SIRT1, LC3-II and Beclin-1) were reversed by treatment with sirtinol or 3-MA. The increased calcium deposition induced by PRP was significantly inhibited in the presence of sirtinol and 3-MA (Fig. 5A). Besides, the osteogenic genes’ expression (COL-I, OPN, Runx2 and OCN) was also remarkably decreased when treated by sirtinol or 3-MA (Fig. 5B).

## Discussion

PDL contains stem/progenitor cell populations, including PDLSCs that allow for the self-renewal and regeneration of periodontal tissues [23]. It has been demonstrated that PDLSCs could undergo chondrogenic, osteogenic and adipogenic differentiation in different inductive conditions [24–26]. To promote the osteogenic differentiation of PDLSCs, there are several emerging methods, including application of erythropoietin, SHED-derived conditioned exposures, and periostin [27–29]. PRP is recognized as a promising material for periodontal regeneration [30, 31]. In the present study, our findings revealed that activated PRP enhanced the cell viability and promoted the osteogenic differentiation and mineralization of hPDLSCs. Our data further showed that autophagy was involved in response to PRP, affecting the osteogenic process in hPDLSCs.

Studies for effects of PRP on mesenchymal stem cells have been reported in several researches. It was found that PRP could promote the cell proliferation and migration of fibroblasts and keratinocytes for adipose-derived mesenchymal stem cells [32]. In another research, Hersant et al. demonstrated that PRP could facilitate the wound healing potential of mesenchymal stem cells [33]. Besides, it was also found that autologous bone marrow mesenchymal stem cells with PRP might promote osteogenesis in a canine model [34]. In the current study, we revealed a higher growth rate of hPDLSCs cultured in culture medium containing a certain concentration range (5–20%) of PRP activated by thrombin than that in medium without PRP in a dose-dependent manner. ALP serves as well-known bone formation marker associated with early osteogenic differentiation [35]. We proceeded to demonstrate the increased ALP activity of hPDLSCs after different concentration of PRP treatment in a dose-dependent manner. This supplement also significantly enhanced the mRNA expression of osteogenic-related factors, such as COL-I, OPN, Runx2 and OCN, compared to the control group. Additionally, higher concentration of activated PRP had stronger effects. Mineralization is a pivotal marker for osteogenic differentiation and bone formation. In our study, the highest mineralization capacity was in 20% PRP, followed by 10% and 5% PRP. Collectively, these results displayed a concentration-dependent effect of activated PRP on the viability and osteogenic differentiation of hPDLSCs in vitro.

Autophagy, also called type II programmed cell death, is a "self-feeding" process involving the degradation of damaged organelles and misfolded proteins in eukaryotes, which plays an important role in physiological and pathological processes during differentiation of MSCs [36–38]. A large quantity of evidence has revealed the chondroprotection induced by PRP was autophagy-dependent [16, 39]. In the present study, we found a marked increase in the expression of SIRT1 and Beclin-1 and the ratio of LC3-II/LC3-I in hPDLSCs incubated in the presence of PRP in a dose-dependent manner. And inhibition of SIRT3 by sirtinol or suppression of autophagy by 3-MA significantly reversed the cell viability and osteogenesis enhanced by PRP, suggesting that autophagy might be an underlying mechanism for PRP-mediated osteogenic differentiation in hPDLSCs. Besides, since both sirtinol and 3-MA inhibit autophagy, and the inhibition of autophagy may lead to inhibition of cell viability in stem cells [40, 41], we speculated that the inhibition of cell viability is due to the inhibition of autophagy when treated by them.

In this study, we demonstrated the dose-dependent effects of activated PRP on the osteogenic ability of hPDLSCs. These findings also provided a new perspective for the role of autophagy in PRP-mediated osteogenic differentiation. Therefore, PRP may have the potential to be used in the culture of hPDLSCs for periodontal regeneration therapy.

## Data Availability

All data can be obtained from the manuscript or from request to the author.
